# Neuroplasticity and Motor Rehabilitation in Multiple Sclerosis: A Systematic Review on MRI Markers of Functional and Structural Changes

**DOI:** 10.3389/fnins.2021.707675

**Published:** 2021-10-06

**Authors:** Eleonora Tavazzi, Marta Cazzoli, Alice Pirastru, Valeria Blasi, Marco Rovaris, Niels Bergsland, Francesca Baglio

**Affiliations:** ^1^IRCCS, Fondazione Don Carlo Gnocchi ONLUS, Milan, Italy; ^2^Department of Neurology, Buffalo Neuroimaging Analysis Center, School of Medicine and Biomedical Sciences, University at Buffalo, State University of New York, Buffalo, NY, United States

**Keywords:** multiple sclerosis, rehabilitation, neuroplastic changes, DTI (diffusion tensor imaging), fMRI

## Abstract

**Background:** Motor rehabilitation is routinely used in clinical practice as an effective method to reduce progressive disability gain in multiple sclerosis (MS), but rehabilitation approaches are typically unstandardized, and only few studies have investigated the impact of rehabilitation on brain neuroplasticity.

**Objective:** To summarize and critically analyze studies applying MRI markers of functional connectivity and structural changes to assess the effect of motor rehabilitation on brain neuroplasticity in MS.

**Methods:** Literature search was performed using PubMed and EMBASE, selecting studies having as a subject motor rehabilitation and advanced MRI techniques investigating neuroplasticity in adult patients affected by MS.

**Results:** Seventeen out of 798 papers were selected, of which 5 applied structural MRI (4 diffusion tensor imaging, 1 volumetric measurements), 7 applied functional fMRI (5 task-related fMRI, 2 resting-state fMRI) whereas the remaining 5 applied both structural and functional imaging.

**Discussion:** The considerable data heterogeneity and the small sample sizes characterizing the studies limit interpretation and generalization of the results. Overall, motor rehabilitation promotes clinical improvement, paralleled by positive adaptive brain changes, whose features and extent depend upon different variables, including the type of rehabilitation approach. MRI markers of functional and structural connectivity should be implemented in studies testing the efficacy of motor rehabilitation. They allow for a better understanding of neuroplastic mechanisms underlying rehabilitation-mediated clinical achievements, facilitating the identification of rehabilitation strategies tailored to patients' needs and abilities.

## Introduction

Multiple sclerosis (MS) is the primary cause of non-traumatic neurological disability in young adults (Goodin, 2014). In the last decades, the progressively expanding availability of pharmacological treatments has changed the disease evolution, with a dramatic impact on the inflammatory component of the relapsing-remitting (RR) phase (Comi et al., 2017). This translates into a reduced relapse rate as well as fewer new/active lesions on magnetic resonance imaging (MRI). Unfortunately, though, the available pharmaceutical armament is considerably less efficacious in treating the neurodegenerative aspect of the disease, with a less meaningful impact on disease progression, which in turns leads to a higher MS-related Global Burden of Disease (GBD) (GBD 2016 Multiple Sclerosis Collaborators, 2019). According to the last GBD Study, neurological diseases are the primary cause of disability-adjusted life years (DALYs), and whereas MS-related mortality has significantly decreased in the last decade, age-related DALYs have not changed (GBD 2016 Neurology Collaborators, 2019). Accordingly, morbidity has a stronger impact on GBD, with moderate-to-severe cases accounting for more than 60% of disease burden (GBD 2016 Multiple Sclerosis Collaborators, 2019; Kaufmann et al., 2020). Moreover, identified risk factors explain <10% of DALYs burden in most neurological conditions, including MS (GBD 2016 Multiple Sclerosis Collaborators, 2019). Therefore, the lack of preventive approaches and the limited ability of pharmacological therapies to prevent neurodegeneration provide a compelling need for strategies capable of mitigating progressive disability gain.

It is clear that physical activity in MS has a positive influence, as demonstrated in both experimental and clinical settings (Dalgas et al., 2009; Rossi et al., 2009; Latimer-Cheung et al., 2013). Whereas earlier studies highlighted the beneficial effects of exercise at a peripheral level (i.e., osteoarticular/muscular and cardiovascular systems), (Dalgas et al., 2009; Latimer-Cheung et al., 2013) it has become increasingly clear that physical activity has a major effect on brain reorganization as well (Prakash et al., 2007, 2010; Motl et al., 2015; El-Sayes et al., 2019; Guo et al., 2020; Lozinski and Yong, 2020). Neuroplasticity, intended as the ability of the brain to modify itself at a structural and functional level in response to aging, experiences and environmental stimuli, occurs throughout the lifespan, as evidenced by animal and MRI based-human studies (Maguire et al., 2000; Draganski et al., 2004; Bengtsson et al., 2005; Zatorre et al., 2012; Garthe et al., 2016; Lambert et al., 2019).

**Figure 1:**
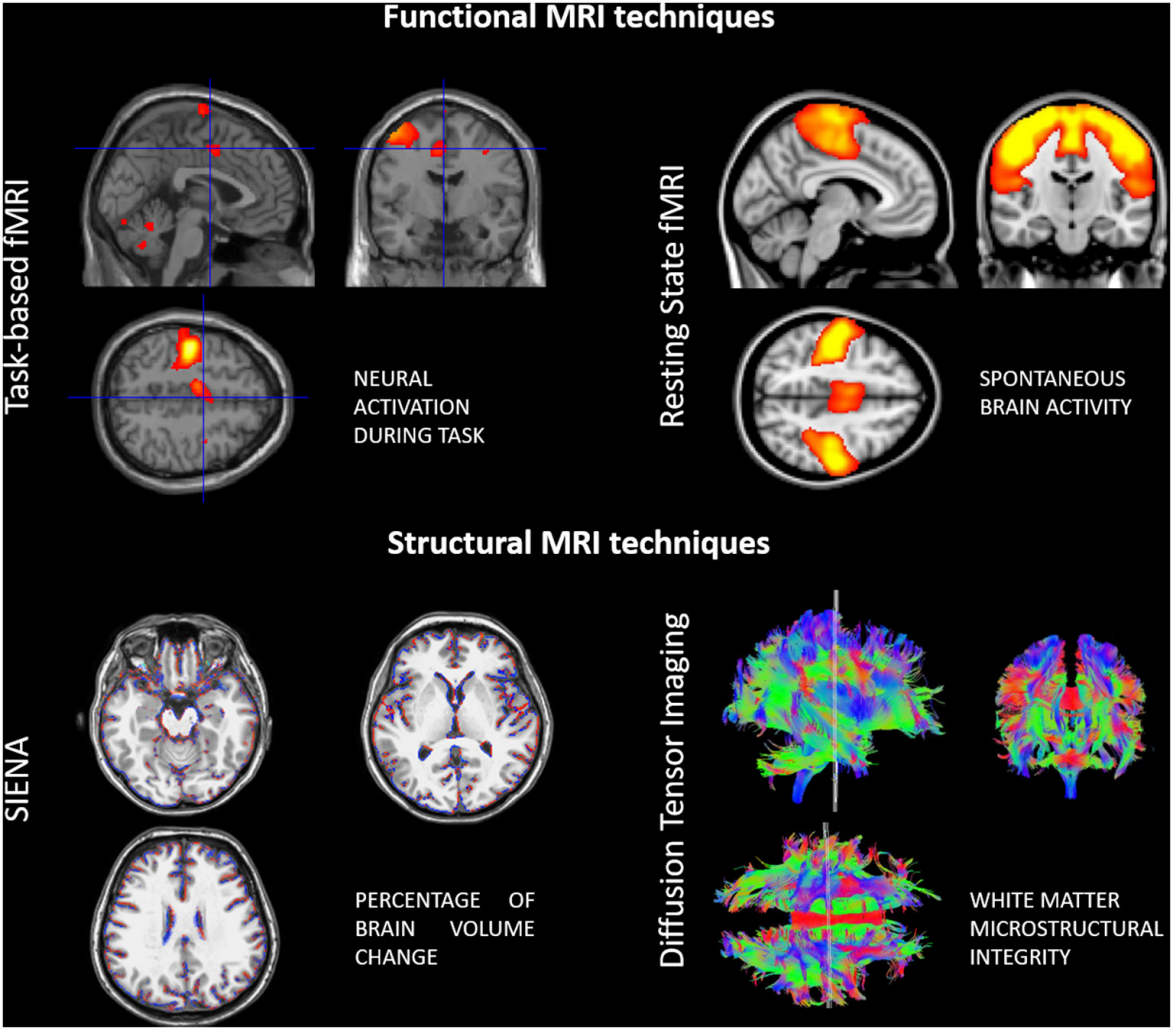
Examples of advanced functional and structural MRI techniques used for assessing neuroplasticity in multiple sclerosis patients.

The more widespread use of advanced imaging techniques, particularly with MRI, has also shed light on the neuroplastic changes that occur in the context of neurological diseases (Figure 1). In particular, in MS, brain tissue repair mechanisms and functional reorganization, at least initially, counterbalance the effect of the two main pathogenic mechanisms involved in the disease progression, inflammation and neurodegeneration (Koudriavtseva and Mainero, 2016).

Diffusion tensor imaging (DTI) is commonly used to study structural aspects, as DTI-derived markers [e.g., mean diffusivity (MD), axial diffusivity (AD), radial diffusivity (RD), and fractional anisotropy (FA)] reflect water mobility within the tissue and along fibers. Volumetric measures of the gray and white matter facilitate the quantification of structural changes over time as well. With functional MRI (fMRI) one can investigate regional or global brain activity, based on blood-oxygenation level dependent (BOLD) signal changes. Previously, fMRI studies were focused on brain activation in response to specific tasks executed by the patients during MRI acquisition (e.g., motor, visual, cognitive). More recently though, resting-state fMRI (rs-fMRI) has become increasingly popular as studying connectivity between different brain areas in resting conditions is possible. This overcomes limitations related to task-execution (standardization of movements in terms of rate and amplitude, possible confounding factors related movement) and also allows the exploration of functional interactions between different brain areas, usually integrated in so-called networks (Tahedl et al., 2018).

Advanced imaging has also been used to study physical activity, with consistent results of a positive effect of exercise on structural changes and functional connectivity (FC) (Prakash et al., 2011) as well as to evaluate the efficacy of neurorehabilitation (Tavazzi et al., 2018).

Structural changes described after rehabilitation treatment include remyelination, neuroaxonal regeneration, neuronal sprouting, synaptogenesis, whereas functional changes reflect adaptive network rearrangements. The net effect is thought to compensate for damaged tissue (Plautz et al., 2000). Importantly though, these processes cannot be studied at the cellular level with MRI, given its much coarser spatial resolution. Moreover, disentangling the role of disparate mechanisms acting simultaneously is challenging. Nevertheless, advanced MRI can still facilitate the study of structural and functional responses in the brain with respect to ongoing tissue damage (Tavazzi et al., 2020).

Despite the fact that rehabilitation is widely utilized for people with MS (pwMS), especially in mid-to-late disease stages, the lack of robust markers, together with the use of different variables (rehabilitative settings, as well as type, frequency, and duration of treatment) make literature studies difficult to compare.

The role of neurorehabilitation on disability accrual and functional impairment is potentially of great clinical relevance, and a better understanding of the underlying mechanisms related to neuroplasticity would have several benefits. First, it would allow for the creation of a more standardized and reproducible process regarding the screening and evaluation of patients eligible for rehabilitation, based not only on clinical but MRI criteria as well. Second, it would facilitate the selection of patients more suitable for rehabilitation intervention. Third, rehabilitative approaches could be tailored to the individual, according to the degrees of physical impairment and tissue injury.

With this background, the aim of this systematic review is to summarize and critically analyze literature data on MRI markers of FC and structural changes (SC) in pwMS undergoing motor rehabilitation.

## Methods

### PICOS Eligibility Criteria

#### Participants

The only eligibility criterion was the recruitment of adult patients (≥18 years old) affected by MS. All disease phenotypes (relapsing-remitting MS-RRMS; progressive MS-PMS) were considered eligible.

#### Interventions

Studies applying quantitative non-conventional MRI techniques on pwMS undergoing motor rehabilitation, both physical (physical exercise, resistance training, aerobic exercise, balance training, endurance training, action-observation therapy, motor rehabilitation using robotic devices for upper or lower limbs) and virtual (virtual reality) were selected. As such, studies reporting MRI results after a single rehabilitative session or studies applying MRI to the study of a specific task that was not part of a rehabilitative treatment were excluded. Moreover, studies using exclusively brain stimulation to enhance brain plasticity, not associated with motor rehabilitation, were excluded. Studies on cognitive rehabilitation of any sort, or studies aiming at improving cognitive functions, were excluded. Finally, treatment was considered “active” when the patient themselves performed the intended action while “passive” was defined as a treatment applied to the patient (e.g., a physiotherapist moving the patient's limb).

#### Comparisons

Both studies with a group of treated patients (i.e., patients undergoing motor rehabilitation of any sort) and a control group of patients not undergoing any treatment, studies comparing groups of patients undergoing different rehabilitative treatments and studies comparing patients undergoing rehabilitation and healthy subjects.

#### Outcomes

The outcome considered was the evaluation of motor rehabilitation on surrogate MRI markers representative of FC and/or SC. Most studies had two time points (e.g., baseline-before rehabilitation initiation-, and post-treatment), only few described a third time point, usually planned 1 month after the end of the rehabilitative treatment, or, in the case of randomized cross-over trials, performed before the 2 groups switched treatment.

#### Study Designs

Peer-reviewed Randomized and non-Randomized Controlled Trials including ≥5 subjects, and case studies were included in the analysis. Conference proceedings, reviews, book chapters, case reports and editorials were excluded.

### Information Sources, Search, and Study Selection

Literature search was conducted using MEDLINE (via PubMed), Cochrane Central Register of Controlled Trials (CENTRAL), and EMBASE from inception to November 3rd 2020. The MeSH terms “multiple sclerosis” AND (“rehabilitation” OR “physiotherapy” OR “exercise” OR “virtual reality” OR “robotics”) AND (“MRI” OR “brain plasticity” OR “connectivity”). Papers written in languages other than English were excluded. References from the selected articles were then screened for further records. Three researchers (ET, NB, MC) independently assessed the selected articles to evaluate their eligibility, and disagreements were solved by discussion.

### Data Extraction

For each study, the study design, number of subjects, rehabilitative setting (e.g., inpatient, outpatient, physiotherapist-supervised, home-based), MRI markers pre- and post-intervention were extracted and reported.

### Testex Evaluation for Randomized Clinical Trials

The included RCTs were evaluated according to the Tool for the assessment of Study qualiTy and reporting in Exercise (TESTEX) scale, specifically developed to evaluate rehabilitative studies both in terms of study quality and reporting. The TESTEX scale consists of 12 criteria and a full scoring of 15 points (Smart et al., 2015). According to the scoring the studies were classified as “high quality” (12–15 points), “good quality” (7–11 points), or “low quality” (6 points or less) as previously suggested (Batalik et al., 2021). In the event that the clinical and the neuroimaging data were reported in separate manuscripts, details were extracted from the associated article that reported a complete description of the protocol.

The current review is reported according to PRISMA (Preferred Reporting Items for Systematic Reviews and Meta-Analyses) criteria (Page et al., 2021). The protocol for the review was not registered before the literature search began.

## Results

**Figure 2:**
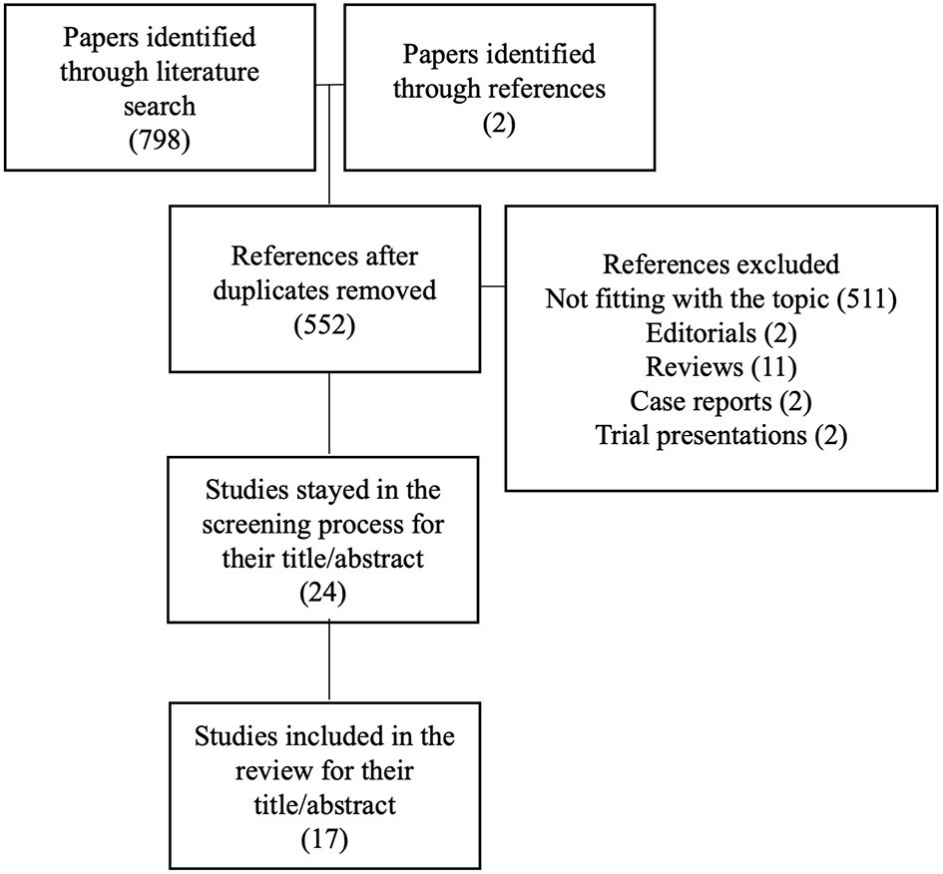
Flow-chart of selection process for the papers included in the systematic review.

The literature search retrieved, as of November 3rd, 2020, 798 papers using the abovementioned MeSH terms, to which we added 2 papers retrieved from references. Seven-hundred and eighty-three papers were eliminated for the following reasons: duplicates (248), not fitting with the topic of the review after reading the title/abstract (511) or the entire manuscript (7), editorials or opinion articles (2), reviews (11), case reports (2), papers reporting only information on planned trials (2). A total of 17 papers are discussed in details in this review. The flow chart summarizing the selection process is depicted in Figure 2.

### Characteristics of the Studies

The key features of the studies are reported in Table 1 for clinical and rehabilitation aspects. Table 2 describes the imaging characteristics. Table 3 provides the TESTEX evaluations of the included studies.

**Table 1:** Clinical and experimental characteristics of the studies.

**References**	**Study design**	**Subjects**	**Experimental treatment setting**	**Control treatment setting**	**Session duration and frequency**	**Clinical outcomes**
**Upper limb rehabilitation**						
Rocca et al. (2019)	RCT	Int = 20 MS, 23 HC Con = 21 MS, 23 HC	Physiotherapist supervised sessions comprising 10 min of right upper limb passive mobilization + 30 min AOT (viewing videos and executing “daily life right hand and arm actions”)	Physiotherapist supervised sessions comprising 10 min of right upper limb passive mobilization + 30 min viewing videos on “inanimate landscapes ” and executing “daily life right hand and arm actions”	HC = 40 min MS = 80 min 2 weeks (5 days/week)	Hand muscle strength (**Jamar**^†^ and Pinch dynamometer); 9HPT; Functional independent measure (1–7); **PASAT-3**; Finger Tapping Frequency
Barghi et al. (2018)	RCT	Int = 10 Con = 10	Outpatient and home-based settings. The CIMT session comprised intensive training of the most affected arm, training by the behavioral technique termed shaping, transfer of behavioral procedure to improve everyday life situation and reducing the use of the less affected arm by wearing of heavily padded restraining mitt for 90% of waking hour	Outpatient and home-based settings. The CAM session consisted of relaxation exercise, aquatic therapy, massage, music therapy and yoga	210 min 2 weeks (5 days/week)	**Motor Activity Log**^†^**;** Wolf Motor Function Test
Bonzano et al. (2014)	RCT	Int = 15 Con = 15	Physiotherapist supervised sessions of active task-oriented training to improve proprioceptive sensibility, muscle strength, stability, and coordination. The percentage of treatment time involving bimanual rather than unilateral tasks increased from 6th to 12th sessions to reach 100%	Physiotherapist-delivered sessions consisting of passive mobilization of upper limbs joints. The same scheme of the active treatment was used for unilateral and bilateral mobilization	60 min 8 weeks (3 days/week)	**ARAT**; **9HPT**; **GRIP**; With the use of an engineered glove finger motor performance accuracy was quantified in terms of **movement rate** at spontaneous (SV) and maximum velocity **(MV)** and (for bimanual task) inter hand interval (**IHI**^†^**)** as an index of bimanual coordination
Bonzano et al. (2019)	RCT	Int = 15 Con = 15	Physiotherapist supervised sessions of active task-oriented training to improve proprioceptive sensibility, muscle strength, stability and coordination. The percentage of treatment time involving bimanual rather than unilateral tasks increased from 6th to 12th sessions to reach 100%	Physiotherapist-delivered sessions consisting of passive mobilization of upper limbs joints. The same scheme of the active treatment was used for unilateral and bilateral mobilization	60 min 8 weeks (3 days/week)	**GRIP**; ARAT; **9HPT**
Boffa et al. (2020)	RCT	Int = 13 Cont = 13	Physiotherapist supervised sessions of active task-oriented training to restore or acquire skills. Unimanual and bimanual tasks were differently weighted in each session and the percentage of time dedicate to bimanual tasks increased from the 7th to the 36th sessions to reach 100%	Physiotherapist-delivered sessions consisting of passive mobilization of upper limbs. The same scheme of the active treatment was used for unilateral and bilateral mobilization	60 min 9 weeks (2 days/week)	EDSS; ARAT; 9HPT; ABILHAND scale; **MFIS (total and physical subscales)**^†^; With the use of an engineered glove finger motor performance accuracy was quantified in terms of movement rate at spontaneous (**SV**) and maximum velocity (**MV**^†^) and (for bimanual task) inter hand interval (**IHI**^†^) as an index of bimanual coordination
**Lower limb rehabilitation**						
Stellmann et al. (2020)	RCT	Int = 34 MS Con = 34 MS	Outpatient setting. Aerobic exercise involving the use of a bicycle ergometer with pedaling rate tailored on the level of physical fitness of each patient	No training (waitlist)	10–100 min 12 weeks (3 days/week)	Primary outcome: VLMT Secondary outcome: EDSS; SDMT; PASAT; Phasic alertness; Tonic alertness; Digits forwards; T25FW; 6MWT; **9HPT**; VO2_peak; VO2 max/kg
Tavazzi et al. (2018)^§^	RCT	Int = 26	Inpatient setting consisting of exercises for global physical functioning and either endurance or progressive resistance training Resistance training was administered using elastic bands and weights. The endurance training involved the use of a treadmill to improve balance and gait adaptability	–	60–90 min 4 weeks (5 days/week)	EDSS; **2MWT**; T25FW; **BBS**; Dynamic gait index; MFIS; MS walking scale-12 items
Kjolhede et al. (2018)^§^	RCT	Int = 17 Con = 12	Physiotherapist supervised sessions of progressive resistance training consisting of 4 lower body and 2 upper body exercises. The intensity (number of repetitions) of exercises increased during the weeks	No training (waitlist)	60–90 min Duration of the sessions increased during the weeks. 24 weeks (2 days/week)	**EDSS**; MSFC: **Total**^†^ and **T25FW**^†^, 9HPT^†^, PASAT; MSIS (physical and psychological subscales); 2MWT; 5-Time Sit-To-Stand Test; muscle strength (**maximal voluntary contractions**)^†^
Ibrahim et al. (2011)	CC	Int = 11 MS HC = 11	Physiotherapist supervised sessions of facilitation physiotherapy comprising sensorimotor stimuli applied repetitively in standard position and motor functions	–	120 min 8 weeks (2 days/week)	**PASAT-3**; EDSS
Prosperini et al. (2014)^§^	RCT	Int = 34 MS (17–17 cross-over) HC = 15	Home based balance training performed using a video game balance board system.	–	30 min 12 weeks (1 day/week)	Force platform–based measures of static standing balance^†^; 4SST; T25FW^†^; MSIS-29^†^
Fling et al. (2019)	RCT	Int = 20 Con = 20	Outpatient setting. The patient underwent an Assistive Device Selection, Training, and Education Program (ADSTEP) consisting of walking training. Participants performed progressive task-oriented training walking with an aid-device under varying conditions	No treatment (waitlist)	40 min 2 weeks (1 day/week)	Primary: Self-Reported Falls Secondary: TUG; 2MWT; T25FW; 4SST; **International Physical Activity Questionnaire**^†^; Quebec User Evaluation of Satisfaction with Assistive Technologies; Multiple Sclerosis Walking Scale-12; Activities-Specific Balance Confidence Scale; MSIS-29
**Multimodal rehabilitation**						
Rasova et al. (2005)	CC	Int = 17 MS Con = 11 MS HC = 13	Outpatient setting. Sessions of “rehabilitative eclectic therapy based on principle of sensorimotor learning and adaptation”	No treatment	60 min 8 weeks (2 days/week)	**T25FW**^†^; **9HPT**^†^; **PASAT-3**^†^; **PR**^†^**; MFIS**^†^; **BDIS**^†^; BI; environmental status scale; **MS QoL**
Prochazkova et al. (2020)	RCT	Int = 20 MS Con = 18 MS HC = 42	Outpatient setting physiotherapist supervised consisting of experimental neuro-proprioceptive facilitation, inhibition intervention: Motor program activating therapy that combines proprioceptive, tactile, visual and auditory stimuli	Outpatient setting consisting of standard neuro-proprioceptive facilitation, inhibition intervention: Vojta reflex locomotion with stimulation of “initiation zone”	60 min 8 weeks (2 days/week)	Primary outcome: MRI data Secondary outcome: 9HPT; PASAT; BBS; T25FW; TUG
Rasova et al. (2015)	CS	Int = 12	Outpatient setting physiotherapist supervised sessions of experimental neuro-proprioceptive facilitation, inhibition intervention: Motor program activating therapy that combines proprioceptive, tactile, visual and auditory stimuli	–	60 min 8 weeks (2 days/week)	Motricity Index; **Modified Ashworth Scale**; BBS; **Tremor**; **dysdiadochokinesis**; **dysmetria**; PASAT-3; **9HPT**; **T25FW**
Akbar et al. (2020)	Semi-RCT	Int = 5 Con = 5	Mostly home-based training in combination with outpatient setting The progressive resistance training involved exercise targeting large muscle groups of upper and lower limbs. The intensity/difficulty of exercises increased during the training session.	Mostly home-based training in combination with outpatient setting Stretching sessions developed following the “Stretching for people with MS” manual.	120 min 16 weeks (3 days/week)	MFIS^†^ (total score and subscales for physical, cognitive and psychosocial impact); T25FW; Godin Leisure-Time Exercise Questionnaire; GRIP
Zuber et al. (2020)	Longitudinal PG	Int = 24 MS HC = 24	Inpatient setting. Personalized multidisciplinary rehabilitation	–	100 min 3 weeks program mean duration: 16.6 ± 3.2 days (min = 10, max = 25.5 days) Session mean duration: 46.1 ± 15.3 h (min = 26.5, max = 80.6 h)	**PASAT**^†^; SDMT; **digit span**^†^ (WAIS-IV); Corsi Block Tapping Test; health-related QoL questionnaire (**SF-12**)^†^;**FSMC motor component**; **9HPT**^†^; **T25FW**
Peran et al. (2020)	SCCS	Int = 26	Inpatient setting. Personalized multidisciplinary rehabilitation which could include: physical therapy, occupational therapy, hydrokinetic therapy, cognitive therapy, therapies for speech, swallowing, respiration and ocular movements	–	135 min 10 weeks (6 days/week)	EDSS; **MSIS-29**; FSS; **BI**; BDIS; **RMI**

*2/6 MWT, 2/6 min walking test; 4SST, 4-step square test; 9HPT, 9-hole peg test; AOT, action observation training; ARAT, action research arm test; BBS, Berg balance scale; BDIS, Beck depression inventory score; BI, Barthel Index; CAM, Complementary and Alternative Medicine; CC, case control; CIMT, constraint induced movement therapy; Con, control group; EDSS, Expanded Disability Status Scale; FSMC, fatigue scale for motor and cognitive function; FSS, Krupp's Fatigue Severity Scale; GRIP, grip strength by a dynamometer; HC, healthy controls; Int, intervention group; MS, multiple sclerosis; MFIS, Modified fatigue impact scale; MSIS, MS impact scale; PASAT, paced auditory serial addition test; PG, parallel group; PR, postural reaction; QoL, Quality of Life; RCT, randomized controlled trial; RMI, Rivermead Mobility Index; SCCS-self-controlled case series; SDMT, symbol digit modality test; TUG, Timed up and go; T25FW, timed 25 foot walk; VLMT, verbal learning and memory test*.

*In bold character are reported the outcome measures with a significant post-vs. pre-treatment effect*.

† *Significant effect for the interventional group compared to the control group*.

§ *The study has an additional timepoint, after the end of the rehabilitation treatment or during it, in randomized crossover trials*.

*Subjects are reported as follows: in case of studies comparing the efficacy of an experimental rehabilitation treatment with conventional physiotherapy, or with no physical activity of any sort, the first group is indicated as “Int” (intervention) and the second as “Con” (control)*.

**Table 2:** MRI characteristic of the studies and significant results.

**References**	**Subjects included in the MRI study**	**MR sequences**	**Post-processing [MRI markers]**	**MRI significant results+**	**Brain area of observed changes**
Rocca et al. (2019)	Int = 20 MS, 23 HC Con = 21 MS, HC = 23	3D T1 TFE	VBM, TBM [GM volumes]	Int MS vs. Con MS:↑GM volume ↓GM volume	L-MOG, L-SFG, R-IFG SMA
				Int MS: correlation between clinical and volumes	[R-IFG, R-SFG]
				Positive correlation: [R-finger tapping, FIM, L Jamar, R Jamar, PASAT] Negative correlation: [PASAT]	[R-SMA]
		DWI	TBSS [FA, MD, RD, AD]	No results	
		RS-fMRI	Seed-based analyses [FC indices]	Int MS vs. Con MS: ↑RS FC Int MS: positive correlation with PASAT	L/R-cerebellum, R-IFG, R-calcarine sulcus Motor and MNS network
		Task-fMRI (manipulation task)	GLM analyses [BOLD maps]	Int MS vs. Con MS: ↑activation (R hand manipulation) ↑activation (L hand manipulation)	R/L-IFG, L-insula L-FG, R-SFG
				Int MS correlations: between clinical and fMRI maps of R hand manipulation [R-finger Tapping, R-Jamar, PASAT]	R/L-IFG, L-insula, L-angular, L-MTG
				Between clinical and fMRI maps of L hand manipulation [PASAT]	R-SFG
Barghi et al. (2018)	Int = 10 Con = 10	DWI	TBSS [FA, MD, RD, AD]	↑FA	PCC, SOG
				↑AD	STG
				↓RD, MD	CST
				Int: correlation between FA and motor improvement	CC and SOG
Bonzano et al. (2014)	Int = 15 Con = 15	DWI	DTI [FA, MD, RD, AD]	↓FA	CC, CST
				↑RD	CC, CST
Bonzano et al. (2019)	Int = 15 Con = 15	Task-fMRI (finger tapping)	GLM analyses [BOLD maps]	↑activation	R-cerebellum, L-pre/post-central Gy, L-insula
Boffa et al. (2020)	Int = 13 Con = 13	DWI	DTI	No results	–
		RS-fMRI	Seed-based analysis [FC indices]	↑FC	bilateral thalami and R-cerebellum; cerebellar vermis and R-insula; cerebellar vermis and RSTG
				Int: Correlation between clinical and FC [IHI-SV, IHI-MV]	R-M1 and R-S1; R/L thalami and R/L IFG; R/L-S1 and L-supramarginal Gy
Stellmann et al. (2020)	Int = 27 MS Con = 30 MS HC = 30	DWI	Graph based analysis structural connectomics [FA]	↑global topology independent structural connectivity (k slope index).	–
		RS-fMRI	Graph based analysis functional connectomics [FC indices]	↑global topology independent FC (k slope index)	–
Tavazzi et al. (2018)	Int = 26	DWI	TBSS [FA, MD, RD, AD]	No results	–
		Task-fMRI (plantar dorsiflexion)	GLM analyses [BOLD maps]	↓extent of scattered task-based activation	Motor and premotor areas
		RS-fMRI	ICA [FC indices]	↑resting FC	pre/post-central Gy bilaterally
Akbar et al. (2020)	Int = 5 Con = 5	RS-fMRI	Seed-based analysis [FC indices]	↑FC	Between caudate and L inferior parietal region, bilateral inferior frontal regions, L middle frontal region, and R insula
				Int: Direct correlation between FC and physical activity level (MFIS)	Caudate, L inferior parietal
Kjolhede et al. (2018)	Int = 17 Con = 12	MPRAGE	GBV, PBVC [GM volumetry, thickness]	↑cortical thickness	Anterior cingulate Gy, temporal pole, orbital Su, and inferior temporal Su following experimental treatment
				Int: correlation between thickness [changes] and clinical data [muscle strength, 5-STS, MSIS, EDSS]	AC-Gy, AC-Su, Orbital H-shaped Su and Temporal pole
Ibrahim et al. (2011)	Int = 11 MS HC = 11	DWI	DTI [FA, MD, RD, AD]	↑FA and ↓MD, ↓RD	CC
Prosperini et al. (2014)	Int = 27	DWI	DTI, Streamline tractography [FA, MD, RD, AD]	↑↓ FA, RD	Bilateral superior cerebellar peduncle
				Int: correlation between FA/RD and postural sway	Bilateral superior cerebellar peduncles
Fling et al. (2019)	Int = 14	RS-fMRI	ROI-based approach [FC indices]	↑ FC	Between the R SMA and the R S1 and between the L SMA and the L S1 as well as the L putamen
				↓FC	Between the SMA and multiple regions in the cerebellum including the R Crus I and bilateral Crus II regions
Rasova et al. (2005)	Int = 17 MS Con = 11 MS HC = 13	Task-fMRI (visually cued pinch grasp)	GLM analyses [BOLD maps]	No results	–
Prochazkova et al. (2020)	Int = 18 MS Con = 17 MS HC = 42	Task-fMRI (video with first person view of dynamic and static scenes)	GLM analyses [BOLD maps]	↑activation	Cerebellum and R frontal lobe
				Int + Con: Positive correlation with a composite clinical index (based on 9HPT, PASAT, BBS, T25FW, and TUG)	Cerebellum, SMA, and premotor area
Rasova et al. (2015)	Int=12	DWI	DTI [FA, MD, RD, AD]	↑FA and ↓MD	CC
		Task-fMRI (flex/ext of joints of the hand)	EC analyses [BOLD maps]	↓EC (trend only)	Between SMA and bilateral M1
Zuber et al. (2020)	Int = 24 MS HC = 24	Task-fMRI (Motor Sequence Learning)	GLM analyses [BOLD maps]	↓ motor activation	L cerebellum and R prefrontal lobe
Peran et al. (2020)	Int = 26	Task-fMRI (MSoA, MimA, PassM)	GLM analyses [BOLD maps]	↓activation	Inferior frontal Gy, middle frontal Gy, inferior and superior parietal lobules, pre-SMA, lateral occipital cortex, thalamus
				↑activation	AC, Inferior orbital Gy, middle temporal Gy, lingual Gy, calcarine cortex, cuneus and precuneus, middle cingulum, M1 and S1

*5-STS, 5x sit-to-stand test; 9HPT, 9-hole peg test; AC, anterior cingulum; AD, axial diffusivity; BBS, Berg balance scale; BOLD, blood oxygenation level dependent; CST, corticospinal tract; DTI, diffusion tensor imaging; DWI, diffusion weighted imaging; EC, effective connectivity; EDSS, Expanded Disability Status Scale; FA, fractional anisotropy; FC, functional connectivity; FIM, functional independent measure; fMRI, functional magnetic resonance imaging; Gy, gyrus; GBV, global brain volume; GLM, general linear model; GM, gray matter; HC, healthy control group; ICA, independent component analysis; IFG, inferior frontal gyrus; IHI-MV, inter-hand interval maximal velocity; IHI-SV, inter-hand interval spontaneous velocity; Int, intervention group; M1, primary motor cortex; MD, mean diffusivity; MimA, miming of action; MFIS, Modified fatigue impact scale; MNS, Mirror Neuron System; MSIS, multiple sclerosis impact scale; MSoA, mental simulation of action; PASAT, paced auditory serial addition test; PassM, Passive Movement; PBVC, percentage brain volume change; pCC, posterior part of the Corpus Callosum; RD, radial diffusivity; R-Jamar, right hand muscle strength with Jamar hand dynamometer; RS-fMRI, resting state functional magnetic resonance imaging; Su, sulcus; S1, primary somatosensory cortex; SDMT, symbol digit modality test; SFG, superior frontal gyrus; SMA, supplementary motor area; T25FW, timed 25 foot walk; TBSS, tract-based spatial statistic; TFE, turbo field echo; TUG, Timed up and go; VLMT, verbal learning and memory test; WM, white matter. ↑ means increased; ↓ means decreased*.

**Table 3:** TESTEX evaluation.

	**Scores**	**Rocca et al. (2019)**	**Barghi et al. (2018)^§^**	**Bonzano et al. (2014)**	**Bonzano et al. (2019)^§^**	**Boffa et al. (2020)**	**Stellmann et al. (2020)^§^**	**Tavazzi et al. (2018)**	**Kjolhede et al. (2018)^§^**	**Prosperini et al. (2014)**	**Fling et al. (2019)^#^**	**Prochazkova et al. (2020) ^§^**
Study quality	Eligibility criteria	1/1	1/1	1/1	1/1	1/1	1/1	1/1	1/1	1/1	1/1	1/1
	Randomization	1/1	1/1	1/1	1/1	0/1	1/1	0/1	1/1	1/1	0/1	1/1
	Allocation concealment	0/1	0/1	0/1	0/1	0/1	1/1	0/1	1/1	0/1	1/1	0/1
	Similarity at baseline	1/1	1/1	1/1	1/1	0/1	1/1	NA/1	1/1	0/1	1/1	0/1
	Blinding of assessors	1/1	0/1	1/1^°^	1/1	1/1^°^	1/1	1/1	0/1	1/1^°^	1/1	0/1^%^
Study reporting	Outcome measures (85% patients)	1/3	2/3	1/3	2/3	1/3	2/3	2/3	2/3	2/3	2/3	1/3
	Intention to treat	0/1	0/1	0/1	0/1	0/1	1/1	0/1	1/1	0/1	1/1	0/1
	Between group statistical comparison	2/2	1/2	2/2	1/2	2/2	2/2	NA/2	2/2	1/2	2/2	2/2
	Point measure and measure of variability	1/1	0/1	1/1	0/1	1/1	0/1	1/1	1/1	1/1	1/1	1/1
	Activity monitoring in the control group^**^	1/1	1/1	1/1	1/1	1/1	0/1	0/1	0/1	0/1	0/1	1/1
	Increased exercise intensity	0/1	1/1	0/1	0/1	1/1	1/1	1/1	1/1	1/1	1/1	1/1
	Exercise parameters	1/1	1/1	1/1	1/1	1/1	1/1	1/1	1/1	1/1	1/1	1/1
	Total score	10/15^*^	8/15^*^	10/15	9/15	9/15^*^	12/15^*^	7/15	12/15^*^	9/15^*^	12/15	9/15

*NA, not applicable*.

* *Study characteristics were retrieved from reference protocol trials or previous/pilot studies from the same group*.

** *The point was assigned also for studies in which the physiotherapy of the control group was either delivered (as in the case of passive mobilization training), or supervised by a physiotherapist. For studies including waitlisted control groups, we awarded 1 point only if any kind of monitoring was specified (e.g., daily exercise diary)*.

° *Blinding of the MRI evaluators and MRI was listed as primary aim of the study*.

% *Blinding of the MRI evaluators was not reported and MRI was listed as primary aim of the study*.

§ *Registered randomized controlled trial*.

# *Points were determined based on the original registered randomized controlled trial*.

The study designs were as follows: 10 randomized clinical trials (RCT) (Bonzano et al., 2014, 2019; Prosperini et al., 2014; Barghi et al., 2018; Kjolhede et al., 2018; Tavazzi et al., 2018; Rocca et al., 2019; Boffa et al., 2020; Prochazkova et al., 2020; Stellmann et al., 2020), plus 1 clinical RCT where the imaging component involved only one subgroup of the intervention arm (Fling et al., 2019). 7 non-randomized clinical trials [1 semi-RCT (Akbar et al., 2020), 2 case-control studies (CC) (Rasova et al., 2005; Ibrahim et al., 2011), 1 case series (CS) (Rasova et al., 2015), 1 parallel-group (PG) (Zuber et al., 2020), 1 self-controlled case series (SCCS) (Peran et al., 2020)].

With respect to patient characteristics, most studies recruited both RRMS and PMS (Bonzano et al., 2014, 2019; Prosperini et al., 2014; Rasova et al., 2015; Barghi et al., 2018; Tavazzi et al., 2018; Fling et al., 2019; Rocca et al., 2019; Akbar et al., 2020; Prochazkova et al., 2020; Zuber et al., 2020), 2 studies recruited only PMS (Boffa et al., 2020; Peran et al., 2020), 3 studies recruited exclusively RRMS (Ibrahim et al., 2011; Kjolhede et al., 2018; Stellmann et al., 2020), whereas disease phenotype was not reported for 1 study (Rasova et al., 2005).

Regarding the rehabilitative treatment, the setting was as follows: inpatient (IP, 3) (Tavazzi et al., 2018; Peran et al., 2020; Zuber et al., 2020), outpatient (OP, 4) (Rasova et al., 2005; Fling et al., 2019; Prochazkova et al., 2020; Stellmann et al., 2020), physiotherapist supervised without further information on the setting (7) (Ibrahim et al., 2011; Bonzano et al., 2014, 2019; Rasova et al., 2015; Kjolhede et al., 2018; Rocca et al., 2019; Boffa et al., 2020), home-based (1) (Prosperini et al., 2014), OP + home-based (2) (Barghi et al., 2018; Akbar et al., 2020). The mean duration of each rehabilitative session was 85 ± 46 min. The weekly frequency of the physiotherapy sessions ranged from 1 to 6 days/week, and the total duration of the rehabilitative program ranged from 2 to 16 weeks.

MRI was always acquired both before and at the end of the rehabilitative cycle, whereas in 5 studies there was also an additional MRI time-point, either within the cycle (Prosperini et al., 2014; Kjolhede et al., 2018), after 4 weeks (Akbar et al., 2020; Prochazkova et al., 2020), or after 3 months (Tavazzi et al., 2018). Among the 17 selected studies, 5 applied structural MRI [4 diffusion tensor imaging (DTI) (Ibrahim et al., 2011; Bonzano et al., 2014; Prosperini et al., 2014; Barghi et al., 2018), 1 volumetric measurements (Kjolhede et al., 2018)], 7 applied functional fMRI [5 task-related fMRI (Rasova et al., 2005; Bonzano et al., 2019; Peran et al., 2020; Prochazkova et al., 2020; Zuber et al., 2020)], 2 resting-state fMRI (Fling et al., 2019; Akbar et al., 2020) whereas the remaining 5 applied both structural and functional imaging (Rasova et al., 2015; Tavazzi et al., 2018; Rocca et al., 2019; Boffa et al., 2020; Stellmann et al., 2020).

### Studies on Upper Limb Rehabilitation

Five studies described clinical and MRI results after upper limb rehabilitation (Bonzano et al., 2014, 2019; Barghi et al., 2018; Rocca et al., 2019; Boffa et al., 2020). All these studies consistently reported a beneficial clinical effect of motor rehabilitation (Barghi et al., 2018; Rocca et al., 2019; Boffa et al., 2020), although the benefits were not significantly different between the active group and the control group undergoing a passive treatment in two studies (Bonzano et al., 2014, 2019). Moreover, there was an association between improved clinical measures and MRI markers of brain reorganization (Barghi et al., 2018; Rocca et al., 2019; Boffa et al., 2020) although the direct correlation between clinical and MRI parameters was not reported in the studies in which passive treatment was utilized (Bonzano et al., 2014, 2019).

With respect to studies analyzing structural MRI markers, white matter structural changes in response to upper limb rehabilitation were less remarkable than functional rearrangements, being either completely absent and not associated with any clinical or behavioral measures (Rocca et al., 2019; Boffa et al., 2020), or small and confined to the corpus callosum and the cortico-spinal tract (Bonzano et al., 2014; Barghi et al., 2018). In this latter case, one study described also a correlation between improved arm function quantified by the Motor Activity Log and corpus callosum integrity (Barghi et al., 2018).

The only study investigating volumetric gray matter changes showed increased volumes in areas involved in the mirror neuron system (MNS) as a response to action-observation therapy (AOT), which is known to activate mirror neurons (Rocca et al., 2019). Furthermore, the volumetric changes of superior and inferior frontal gyrus were significantly associated with the main clinical measures of strength (Jamar dynamometers), hand dexterity (9-hole peg test), and finger tapping frequency, as well as with cognitive measures of attention and processing speed (Rocca et al., 2019).

Functional changes in response to rehabilitation, on the contrary, are reported in all the studies, with different activation patterns, which largely depended on the specific type of rehabilitation approach: MNS and cerebellum for AOT (Rocca et al., 2019), primary motor area, primary sensorimotor cortex, thalami, and cerebellum for task-oriented therapy (Bonzano et al., 2019; Boffa et al., 2020). Remarkably, in both rs-fMRI and task-related fMRI studies the cerebellum showed increased activation/connectivity with other brain areas. Moreover, Rocca et al. reported significant associations between improved strength and dexterity, measured, respectively, with dynamometers and 9-hole peg test and both functional connectivity as task-related activations of different cortical areas, but mainly of the inferior frontal gyrus (Rocca et al., 2019). Boffa et al. described significant associations between lower interhand interval at spontaneous and maximum velocity and the functional connectivity between primary motor cortex and primary somatosensory cortex, as well as between thalami and inferior frontal gyrus (Boffa et al., 2020).

### Studies on Lower Limb Rehabilitation

Six studies focused on lower limb rehabilitation, intended as either aerobic exercise or resistance training (Ibrahim et al., 2011; Kjolhede et al., 2018; Tavazzi et al., 2018; Fling et al., 2019; Stellmann et al., 2020), and in one case balance training (Prosperini et al., 2014).

Clinical improvements, when investigated, were related to motor functions (Tavazzi et al., 2018), standing balance (Prosperini et al., 2014), and cognitive performance (Ibrahim et al., 2011).

Two DTI studies reported improved tissue integrity in structures belonging to the motor pathway, such as the corpus callosum (Ibrahim et al., 2011) and superior cerebellar peduncles (Prosperini et al., 2014), although other relevant regions investigated by Prosperini et al. (2014) (e.g., middle and inferior cerebellar peduncles, corpus callosum, corona radiata) did not show any change. In the latter study, improved balance reflected by reduced postural sway was strongly associated with both increased FA and reduced radial diffusivity of superior cerebellar peduncles (Prosperini et al., 2014).

Improved structural integrity reflected by increased white matter FA, together with within-hub structural integration and organization, was also found in the only connectome-based study, paralleled by increased FC and associated with Expanded Disability Status Scale (EDSS) change (Stellmann et al., 2020). On the contrary, another study reported no significant structural changes investigated by means of Tract-Based Spatial Statistics, as well as no associations with clinical parameters (Tavazzi et al., 2018).

All the rs-fMRI studies investigated exclusively region of interests targeted by the specific type of rehabilitation and reported increased FC within the sensorimotor networks (Tavazzi et al., 2018; Fling et al., 2019). The only study applying a connectome-based approach confirmed and expanded previously reported results, describing increased global FC, which was associated with improved structural organization but not with any clinical measures. In one study, the increased connectivity between supplemental motor area (SMA) and sensorimotor network was paralleled by reduced connectivity with the cerebellum (Fling et al., 2019).

### Multimodal Rehabilitation

The last six studies described results from multimodal rehabilitation, intended as exercises focused at improving motor functions of both upper and lower limb, as well as trunk balance (Rasova et al., 2005, 2012, 2015; Akbar et al., 2020; Peran et al., 2020; Prochazkova et al., 2020; Zuber et al., 2020).

The only structural study reported improved tissue integrity represented by increased FA and reduced MD within the corpus callosum, as well as an improvement of several clinical measures, even though the association between clinical and MRI outcomes has not been described.

Task-related fMRI results in multimodal rehabilitation studies are discordant, reporting increased activation of SMA and premotor cortex significantly associated with improved clinical index only in patients clinically improved (Prochazkova et al., 2020), reduced cerebellar and prefrontal activation in the active group paralleling an increased accuracy in the execution of the motor task (Zuber et al., 2020), or no significant changes between active and control group (Rasova et al., 2005, 2012, 2015). Finally, another study showed reduced activation in areas usually involved in the specific task, whereas activation was increased in new cerebral areas. As the efficacy of the rehabilitation intervention was not an aim of the study, clinical measures were not reported (Peran et al., 2020).

One study investigated the effect of progressive resistance training of both upper and lower limbs on both fatigue and caudate connectivity, reporting a significant improvement of the physical and cognitive components of fatigue as well as increased functional connectivity between caudate and several cortical areas (Akbar et al., 2020). Furthermore, the increased functional connectivity between caudate and left inferior parietal lobule was significantly associated with increased physical activity level, and there was a trending correlation between the abovementioned increased FC and a decrease in cognitive fatigue quantified by the modified impact fatigue scale (Akbar et al., 2020).

### Quality Ratings

Detailed information about the TESTEX scoring are reported for each item in Table 3.

Eleven out of 17 included studies were RCT and were evaluated with the TESTEX scale (Smart et al., 2015). Three out of 11 were classified as “high quality” studies (12–15 points), while 8 out of 11 were classified as “good quality” studies (7–11 points). Five out of 11 RCT were registered. One study involved only imaging of 14 patients in the experimental group (Fling et al., 2019), while the larger RCT was published in a different manuscript and was registered.

## Discussion

Motor rehabilitation in MS has a global beneficial effect both subjectively perceived as reduced fatigue, increased motivation and level of physical activity and objectively quantified by several clinical scales. Considering that MRI is a fully integrated tool in the MS-related diagnostic and monitoring processes, the low number of studies applying MRI to evaluate the effect of rehabilitation with respect to the total amount of studies dedicated to rehabilitation in MS is somewhat surprising. Several studies here reported underline the crucial role of MRI markers in the evaluation of rehabilitation effects, even when clinical markers fail to do so (Bonzano et al., 2014, 2019). However, the scarceness of results, together with their heterogeneity, makes it difficult to clearly define the effects of rehabilitation on brain functional and structural changes. Moreover, the role of neurorehabilitation on brain plasticity is challenging, considering that MS is characterized by chronic, ongoing tissue damage, such that changes are happening in tandem with response to inflammation and neurodegeneration. Overall, most of the studies analyzed in this review show that motor rehabilitation has the potential to favorably impact brain neuroplasticity, although many factors, such as individual disease stage and duration, as well as rehabilitation type and duration, influence the type and degree of cerebral response.

### Adaptive vs. Maladaptive Plasticity

Adaptive plasticity has been extensively studied, as the capacity of the brain to structurally and functionally change in response to experiences and environmental stimuli in healthy subjects, as well as in response to tissue injury, with the aim to restore homeostasis (Nava and Roder, 2011). However, changes can also be considered as reflecting so-called maladaptive plasticity, which represents an aberrant modification associated with a poor clinical outcome (Trojan and Pokorny, 1999; Nava and Roder, 2011). Often though, the two mechanisms are closely interrelated and the border between the two phenomena is very narrow. Maladaptive plasticity has been the subject of several studies in motor-related stroke patients (Jang, 2013), where it results in abnormal movement patterns or increased activation of the contralesional motor pathway, thus preventing the recovery of the damaged area. In MS, the distinction between adaptive and maladaptive mechanisms is fraught with challenges in the absence of associated clinical measures, as the underlying tissue damage renders it difficult to interpret changes unequivocally (Laura et al., 2018). Widespread functional brain activation in response to motor or cognitive tasks is generally increased in MS patients with respect to healthy subjects, leading to define it as a compensatory mechanism to preserve a satisfactory clinical status (Tavazzi et al., 2018; Rocca et al., 2019). However, some studies showed that better motor performances were associated with a reduction of diffuse task-related activation after rehabilitation intervention, interpreted as increased synaptic efficiency and recovery of specialized function within the damaged area (Tavazzi et al., 2018; Bonzano et al., 2019; Peran et al., 2020). It must be noted though that other studies reported increased task-related activation and increased FC in association with improved motor functions (Guerrera et al., 2014; Rocca et al., 2019; Akbar et al., 2020; Boffa et al., 2020). This apparent discrepancy confirms that the same plasticity mechanisms can result in opposite clinical outcomes and be considered either adaptive or maladaptive, likely depending on several factors including tissue damage entity and location along with disease stage, among others. It has also to be considered that new sophisticated tools to study global brain connectivity rather than task-related brain activations, such as the connectome-based approach, have shown a very complex hierarchical organization within the brain, consisting of networks and hubs interacting across disparate brain regions. Therefore, a dichotomic distinction of functional rearrangements between adaptive and maladaptive might be overly simplistic in MS, but only future longitudinal studies will clarify this aspect (Schoonheim et al., 2015).

### Upper Limb Rehabilitation

Although walking impairment is frequently considered the most disabling aspect of MS, upper limb dysfunction is frequent and strongly impacts quality of life at multiple levels (Johansson et al., 2007; Bertoni et al., 2015). Results from the studies included in the present review show that neuromotor rehabilitation effectively improves upper limb function, although in two studies there was no significant difference between the active and the passive groups, in terms of clinical outcomes (Bonzano et al., 2014, 2019). This might be due to the fact that passive mobilization can be an effective stimulus on the corresponding sensorimotor cortex, or might be related to the choice of clinical measures that are not necessarily sensitive enough to detect a difference between the two groups. This latter hypothesis seems likely, considering that structural MRI markers were stable over time in the active group but worsened in patients undergoing the passive treatment (Bonzano et al., 2014), and functional MRI showed a trend toward brain activity normalization only in the active group (Bonzano et al., 2019).

Studies investigating structural brain reorganization elicited by motor rehabilitation reported very small changes, if any (Bonzano et al., 2014; Barghi et al., 2018; Rocca et al., 2019; Boffa et al., 2020). Even though structural changes have been reported early after training, the short treatment period and the long disease duration in the aforementioned studies might account for their results, related to an exhaustion of compensatory mechanisms in the brain, thereby preventing meaningful structural reorganization (Filippi et al., 2012; Bonzano et al., 2014).

Functional changes, instead, were described in all the studies, involving different areas mostly depend upon the rehabilitation approach used. However, a common finding of these studies was increased cerebellar connectivity with multiple brain areas. The cerebellum is a key structure involved in several cognitive and motor functions and is associated with multiple cortical areas through afferent and efferent cortico-cerebellar pathways (Stoodley and Schmahmann, 2010; Ruggieri et al., 2020). Recently, FC cerebellar changes were reported for both sensorimotor and cognitive compartments as a consequence of MS-related tissue damage, negatively associated with global and regional disability levels (Pasqua et al., 2020). This latter finding supports the adaptive role of cerebellar FC, highlighted also by the abovementioned results in the rehabilitative setting.

### Lower Limb Rehabilitation

Motor dysfunctions involving the lower limbs are so frequent and relevant that the clinical scale primarily used to quantify physical disability, namely the Expanded Disability Status Scale (EDSS), weights this aspect above all others, and disability milestones are related to the gradual loss of walking autonomy. Therefore, rehabilitation of lower limbs, which includes treatment of weakness, spasticity and imbalance, is a relevant part of the clinical practice.

Most structural studies reported a positive effect of rehabilitation on motor pathway tissue integrity. An association between structural rearrangements in motor-related areas and improvement of clinical measures reflecting motor functions was also described, leading to interpret these changes as beneficially adaptive (Prosperini et al., 2014; Stellmann et al., 2020). The only study that failed to show rehabilitation-mediated structural changes as well as a significant association structural MRI markers and clinical outcomes, was hampered by a small sample size and a high drop-out rate (Tavazzi et al., 2018).

Functional results are concordantly positive, although the analysis of most studies was limited to regions of interests, known to be activated by the chosen rehabilitation approach. However, the connectome-based approach used in one single study showed a significant association between functional and structural reorganization at a global level (Stellmann et al., 2020).

Interestingly, reduced connectivity between SMA and the cerebellum has been reported (Fling et al., 2019). Although this finding, which is the opposite of what previously reported in upper-limb rehabilitation studies, can be ascribed to different aspects related to the rehabilitation approach or patient characteristics, it can also be hypothesized that upper-limb and lower-limb rehabilitation potentiate different pathways, in which the cerebellum can be variably involved. Overall, the association between structural and functional changes mediated by neuromotor rehabilitation and improved clinical outcomes strongly suggests a positive effect of rehabilitation on the brain.

### Multimodal Rehabilitation

As MS is a neurodegenerative disease impacting several multifunctional systems, the consequent disability usually involves all 4 limbs, to a varying degree and mainly in the late stage, as well as the trunk in terms of imbalance and postural lack of control. Some rehabilitative approaches combine exercises for both upper and lower limbs, and so-called multimodal or multidisciplinary rehabilitation includes different types of treatment, such as motor rehabilitation and occupational therapy, with the aim of maintaining everyday life activities. The identification of structural and functional changes occurring in response to these rehabilitative therapies is challenging, due to the multiplicity of brain areas potentially recruited.

Structural brain reorganization in response to multimodal rehabilitation was concordantly reported and associated with improved motor and cognitive performance, suggesting a protective role of rehabilitation in MS-related neurodegeneration (Kjolhede et al., 2018). However, functional data are very heterogeneous both in terms of activation pattern and regions involved.

A general issue with multidisciplinary rehabilitation interventions relates to the fact they are typically tailored to individual patient needs and abilities, and as such vary between different centers and even within subjects of the same group. This aspect, while representing a significant advantage in the clinical practice, becomes a major issue in the research field as it potentially makes it more difficult to identify signals in the data and renders it challenging to compare studies and apply their results in different settings.

### Methodological Considerations and Potential Bias

The design and conduction of studies focused on MRI markers of neuroplasticity in rehabilitation settings present several potential issues that are worth discussing (Table 2). In addition, it is important to consider possible sources of bias in the studies that have been reviewed. Although the techniques utilized in provide quantitative measures, it is difficult to directly compare the results from one study to another due to technical factors. For example, scanning platforms and sequence parameters of the image acquisition vary substantially between studies. Furthermore, it is typically the case that all details describing the specific options used in image post-processing are not fully provided in a given manuscript. Importantly, it may also be the case that the authors explored multiple different processing techniques but only chose to present the “best” results, without mentioning that other avenues had been pursued. To mitigate these sources of biases, authors can choose to register a protocol with their analysis plan before the study even starts such that subsequent findings can be directly linked to the original intent. As of now though, most reported studies have not registered a protocol ahead of time, resulting in a considerable risk of bias and thus preventing one from drawing concrete conclusions from individual studies. Of note, the analysis protocol was not published prior to study initiation for any of the reported studies in this review.

#### Disease Stage

Contrarily to what happens in other neurological conditions such as traumatic brain injury or a first episode of stroke, in which a monophasic event occurs in an otherwise healthy brain, MS is a chronically evolving disease. Therefore, the disease stage, and consequently the balance between the ongoing inflammation and neurodegeneration, is a relevant factor to be considered when analyzing the effect of rehabilitation on brain reorganization. Indeed, inflammation is known to impact neuroplasticity via several mechanisms, interfering with synaptic transmission, neurovascular coupling and signal transmission through non adjacent brain regions (Tomassini et al., 2016). Neurodegeneration is characterized by progressive loss of tissue integrity, with chronic demyelination in both the white and gray matter as well as neuroaxonal damage intrinsically affecting brain plasticity (Ksiazek-Winiarek et al., 2015). Whereas these notions might seem to suggest that late-stage MS patients would not benefit from rehabilitation, it has instead been demonstrated that the brain does not fully exhaust its neuroplastic abilities, even in severely disabled patients (Tomassini et al., 2011, 2012). However, clinical trials evaluating the efficacy of rehabilitation should recruit homogenous populations in terms of disease stage, or at least take into account this factor when interpreting the results. MRI markers of inflammation, such as gadolinium-enhancing lesions or new/enlarging T2-lesions, as well as markers of neurodegeneration, such as volumetric measures quantifying tissue atrophy or DTI parameters, could be relevant information to include, rather than the simple categorization of patients as RRMS or PMS.

Along this line, the presence of disease modifying treatments should also be considered, as it has been showed that a pharmacologically-driven reduction of inflammation parallels restoration of brain plasticity (Tomassini et al., 2016).

#### Control Group

The choice of an appropriate control group is a critical point when aiming to detect changes at a microstructural level (Thomas and Baker, 2013), as well as functional rearrangements, especially when the subject of the study is a chronic disease affecting both white and gray matter.

Comparing MS patients with healthy subjects undergoing the same rehabilitation intervention is not ideal (Ibrahim et al., 2011), as neuroplastic mechanisms depend on the underlying degree of tissue integrity. Even when recruiting MS patients as control groups, the choice of “placebo” treatment needs to be carefully evaluated. The complete absence of treatment (Rasova et al., 2005; Stellmann et al., 2020), as well as a shorter exposure time to physical activity makes it difficult to interpret whether the potential results could be attributed only to increased level of physical activity or be the consequence of brain reorganization. A control group with MS patients undergoing a different type of training, including passive movements, for the same amount of time, represents the best option (Bonzano et al., 2014, 2019; Barghi et al., 2018; Rocca et al., 2019; Akbar et al., 2020; Boffa et al., 2020; Prochazkova et al., 2020).

#### Follow-Up

Only 3 studies reported data on follow-up, which was short-term (4 weeks) in 2 of them (Rasova et al., 2015; Zuber et al., 2020), and 3 months in the remaining one (Tavazzi et al., 2018). Whereas the studies with short-term follow-up reported maintenance of functional (Zuber et al., 2020) and structural (Rasova et al., 2015) changes after rehabilitation, patients reverted to the baseline in the study with longer follow-up (Tavazzi et al., 2018). The persistence of clinical, as well as functional and structural improvements obtained with rehabilitation, is a crucial aspect that needs to be carefully investigated, as the main aim is for pwMS to be able to exploit the benefits of the rehabilitation intervention in a real-life setting. Surely several factors play a relevant role, such as the socio-familial support, psychological state, and environment that allows for the maintenance of a physically active lifestyle (Kever et al., 2021). Interestingly though, whereas neuroplastic abilities are preserved throughout the disease course as abovementioned, retention skills might be impaired in pwMS (Nguemeni et al., 2020), explaining the negative results of the longer-term follow-up study. Only studies with longer follow-ups can help better understand whether the clinical and MRI- related achievements obtained with motor rehabilitation are the mere results of increased physical activity or they reflect a more consolidated process of brain reorganization, and how to favor this second possibility.

#### Other Factors

Some other factors are worth mentioning, as they could hinder rehabilitation-mediated clinical and MRI results or act as confounders.

While investigating the efficacy of motor rehabilitation on brain reorganization, one aspect that should be taken into careful consideration is the overall integrity of the motor pathway. In stroke patients, structural damage of the pyramidal tract, including both neurons belonging to motor areas and axons of the corticospinal tract, considerably influence the type and extent of functional rearrangements after rehabilitation (Hamzei et al., 2006, 2008). In pwMS with moderate-to-severe disability, the likelihood of motor pathway damage is high. Nonetheless to characterize its extent might be useful when trying to explain different types of functional rearrangements, and interpreting structural data (Naismith et al., 2013; Oh et al., 2013; Hubbard et al., 2016).

Fatigue was quantified in 7 out of 17 studies (Rasova et al., 2005; Tavazzi et al., 2018; Akbar et al., 2020; Boffa et al., 2020; Peran et al., 2020; Stellmann et al., 2020; Zuber et al., 2020), and is another element that should be assessed in patients undergoing neuromotor rehabilitation. Fatigue is one of the most common and disabling symptoms affecting pwMS, negatively impacting quality of life and disease evolution (Eizaguirre et al., 2020; Vaughn et al., 2020), as well as reducing overall physical activity. Fatigue has a neuroimaging correlate, being associated with altered FC within the sensorimotor networks, and hyperactivation of different brain regions, such as the primary motor area (Bertoli and Tecchio, 2020). As such, fatigue could interfere with both clinical outcomes and neuroplastic changes mediated by rehabilitation. In the past, pwMS were advised to abstain from exercise to reduce symptoms as fatigue, but it has been demonstrated that, on the contrary, fatigue improves together with endurance in pwMS undergoing regular exercise (Stephens et al., 2019), or rehabilitation. Therefore, fatigue should be regularly assessed in MRI studies testing rehabilitation efficacy, as it could be both a marker of rehabilitation efficacy and a factor that needs to be considered when interpreting clinical and MRI results. A better understanding of the role of fatigue and its functional MRI correlate would lead to the inclusion of rehabilitation strategies specifically aimed at reducing its impact on quality of life in pwMS (Dobryakova et al., 2018; Akbar et al., 2020).

### Future MRI-Rehabilitation Studies

Physical activity is now recommended as a strategy capable of impacting disability accrual in pwMS (Lozinski and Yong, 2020), but some studies described an effect of exercise also on cognition, mediated by neuroplasticity, proving a broad effect of physical exercise that goes beyond clinical motor outcomes and motor-related areas in the brain (Leavitt et al., 2014; Sandroff et al., 2017, 2018). In particular, enriched environmental stimuli result in greater neuroplastic abilities, as evidenced by experimental and human studies (Carey et al., 2005; You et al., 2005; Lambert et al., 2016; Saleh et al., 2017; Wu et al., 2019). Therefore, rehabilitation strategies exploiting virtual reality, robotic devices, and/or dual task approaches need to be further studied and subsequently implemented in clinical practice.

With respect to imaging techniques applied in the study of rehabilitation effects on brain reorganization, tools that simultaneously analyze structural and functional rearrangements of multiple brain areas, based on the hierarchical organization of brain networks, should be favored (Stellmann et al., 2020).

Finally, the wider availability of technological devices makes telerehabilitation an option that needs to be considered, at least complementing therapist-supervised treatments, as it could more easily reach pwMS with limited access to rehabilitation centers in addition to lowering overall MS-related economic burden. Few studies have been done in this context, but none have applied MRI markers to test the intervention efficacy, and the derived evidence is not robust enough to draw any conclusions (Khan et al., 2015). However, it has emerged as a need for pwMS even with mild disability (Remy et al., 2020), and the potential usefulness in more severely disabled patients highlights the need for further studies applying telerehabilitation and including MRI outcomes.

## Summary and Conclusion

MS is a chronic, long-lasting disease affecting mainly young people in whom the maintenance of ambulatory self-sufficiency is crucial for psychological, relational, socio-economic and work-related reasons.

In this complex disease, the individual recovering abilities, in terms of both clinical disability and neuroplasticity, derive from several factors such as ongoing pathogenic mechanisms, disability level, cognitive impairment, as well as socio-economic and familial conditions. Neuromotor rehabilitation is a crucial aspect among all the interventions aimed at preserving quality of life and reducing MS-related GBD. However, studies investigating the effect of rehabilitation on brain reorganization are few and the validity of their results is hampered by the small sample size, potential bias in lack of protocol registration, and the extreme data heterogeneity in terms of patient characteristics, rehabilitation settings, MRI protocol acquisition and post-processing. Conversely, a better standardization of rehabilitation interventions is needed, to facilitate the identification of the appropriate rehabilitation approach for individual patient needs and abilities, and the generalization of results. In this context, MRI markers of structural and functional connectivity represent an important part of this assessment process and should be implemented in the clinical routine as well as in research studies investigating the effect of rehabilitation interventions. Indeed, they provide quantitative, reproducible information on neuroplastic changes underlying clinical achievements, furthering the community's knowledge on the role of rehabilitation modifying disease evolution, and contributing to tailored rehabilitation strategies at an individual level.

Finally, considering that an unequivocal interpretation of structural and functional findings is difficult and further complicated by the ongoing pathological mechanisms of a chronically evolving disease such as MS, associations between MRI markers and clinical/behavioral data should always be presented. This is fundamental for characterizing changes in terms of adaptive or maladaptive responses and facilitates the assessment of success for a given rehabilitative treatment.

## Data Availability Statement

The original contributions presented in the study are included in the article/supplementary material, further inquiries can be directed to the corresponding author/s.

## Author Contributions

ET: literature search, data interpretation, and scientific writing. MC: literature search, tables preparation, and scientific writing. AP: literature search and figure preparation. VB: data interpretation and analysis. MR: scientific writing and critical data analysis. NB: literature search, figure preparation, and scientific writing. FB: scientific supervision. All authors contributed to the article and approved the submitted version.

## Funding

This study was funded by the Italian Ministry of Health (Ricerca Corrente Reti Program- Neurosciences and Neurorehabilitation Network). ET received a scholarship from Crespi Spano Foundation.

## Conflict of Interest

The authors declare that the research was conducted in the absence of any commercial or financial relationships that could be construed as a potential conflict of interest.

## Publisher's Note

All claims expressed in this article are solely those of the authors and do not necessarily represent those of their affiliated organizations, or those of the publisher, the editors and the reviewers. Any product that may be evaluated in this article, or claim that may be made by its manufacturer, is not guaranteed or endorsed by the publisher.
